# Supplementary material for: Childhood overweight and obesity at the start of primary school: External validation of pregnancy and early-life prediction models
Source: PLOS Glob Public Health. 2022 Jun 9;2(6):e0000258. doi: 10.1371/journal.pgph.0000258 (PMC10022097; doi:10.1371/journal.pgph.0000258)
Supplement: Attachment — Submitted filename: Response_to_reviewers_comments.docx [file pgph.0000258.s001.docx]

Responses to reviewers comments

We invite you to submit a revised version of the manuscript that addresses the points raised during the review process.
1. We recommend expanding the description of the model with more details, as well as it's contextualization as suggested by the reviewers.

*Thank you, we have now included a more detailed description of the model (lines 95-109 in the clean and 96-111 in the tracked version) and its contextualisation (lines 76-88 in the clean and 77-89 in the tracked version) as suggested.*
2. We believe that the quality of the work will improve with a little more insight into the strengths and limitations section.

*Thank you, we have included more insight into the strengths and limitations section (lines 293-314 in the clean and 297-318 in the tracked version).*
4. We recommend to raise the issue and elaborate the practical applications of the model.

*We have added a section about the practical application of the model* *(lines 344-356 in the clean and 348-360 in the tracked version).*
5. We also recommend the revision of the text in terms of references to the literature and supplement any gaps.

*We have revised the text in terms of references to the literature as suggested by the reviewers. Details are outlined below in the responses to specific reviewer comments.*

Journal Requirements:

1. Please provide additional details regarding participant consent. In the ethics statement in the Methods and online submission information, please ensure that you have specified what type you obtained (for instance, written or verbal, and if verbal, how it was documented and witnessed).

*Written informed consent was obtained. Details have been included in the methods section (line 124 in the clean and 127 in the tracked version) and online submission information.*

2. Please provide separate figure files in .tif or .eps format only.

*Separate figure files in .tif format have been uploaded as part of the revision.*

3. Please update the completed 'Competing Interests' statement, including any COIs declared by your co-authors. If you have no competing interests to declare, please state "The authors have declared that no competing interests exist". Otherwise please declare all competing interests beginning with the statement "I have read the journal's policy and the authors of this manuscript have the following competing interests:"

*The authors have no competing interests to declare so this has been updated to “The authors have declared that no competing interests exist”.*

Additional Editor Comments (if provided):
We have carefully checked the type of publication pointed out by the Reviewer #2. and due to the fact that the indicated source is an abstract of the conference report, we believe that the requirements of the journal have not been violated and the article may be further processed for publication.

Thats why the first comment of the Reviewer #2. as well as the point 1., and 2. can be ignored.

*Thank you for checking and clarifying.*

Reviewer's Responses to Questions

**Comments to the Author**

Reviewer #1:

Title
Is the model not developed for predicting childhood overweight and obesity up to the age of 2? Therefore, is it applicable to primary school children?
*The model uses data up to the age of 2 years to predict risk of childhood overweight and obesity at the age of 4-5 years (first year of primary school in the UK).*

Abstract
Good. The abstract states the main findings. Could be aided by breaking up the abstract into introduction, methods, results, and conclusion.
*Thank you. The abstract has been drafted in line with PLOS Global Public Health’s author guidelines which states “While the Abstract is conceptually divided into three sections (Background, Methodology/Principal Findings, and Conclusions/Significance), do not apply these distinct headings to the Abstract within the article file.”*

Introduction
Excellent introduction to the utility of risk prediction models. Very minor suggestions:
1. Add a few sentences about the strategies that could be implemented post risk stratification. Without a way to reduce the risk in high-risk populations, there is a possibility this model could pave the way for future discrimination.

*Thank you, we have added consideration of this in the introduction (lines 56-68 in the clean and 57-69 in the tracked version).*

2. A reference is needed for this:
“Weight can be a sensitive topic to raise and our consultation work with practitioners suggests that health professionals would like an ‘objective’ way to stratify risk rather than individualised clinical judgement which feels subjective.”

*A reference has been added for the first part of the sentence (line 54 in the clean and 55 in the tracked version). There is no reference available for our consultation work with practitioners.*

3. A subject needs to be added to this clause:
“as only utilises data that is 63 already being collected”
*Thank you, this has been added (line 74 in the clean and 75 in the tracked version).*

Methods
Excellent methods section. No further improvements to suggest here.
I do have a question, however. By including ethnicity as one of the model predictors, is there a chance that this model will become redundant in future years as the sociocultural factors that link ethnicity to childhood BMI change? Does it also limit the model’s utility to the UK as the cultural factors surrounding ethnicity will be different in different parts of the world? The model's geographical limitation to the UK and temporal usability also applies for the choice of options for maternal educational attainment and maternal first language.
*Thank you. Prediction models are most relevant in the population used to develop the data. Validation is required before it can be used in other populations and settings. This external validation study shows that it is relevant within an unrelated population in the UK. Use in other countries or settings will require validation before use to ensure it works in the context. However, this does provide a base model that can be recalibrated to other populations if needed, therefore the work is not started from scratch if applied to populations in other countries. We have also previously conducted a systematic review of prediction models of childhood obesity which included studies from outside the UK and this has helped inform our analysis. We hope our models also inform further prediction models. Current associations need to be included in the model to ensure validity. If factors linked to current associations change, the model can be updated/recalibrated to ensure it remains relevant.*

Results
Interesting results.
I note the higher prevalence of childhood overweight and obesity in the group that has been grouped by the maternal ethnicity of “Black/African/Caribbean”. Is there a risk we’re reducing very heterogenous cultures into a monolith? For examples trends such as encouraging children to finish their whole bottle of milk or propping the bottle up are only present in certain cultures within this communities that have been grouped together. This model risks stereotyping everyone from said community.

*We agree that this is a broad ethnic group and variations in cultural practices within different groups may be lost. However, as ethnicity is one of several factors used to identify those at risk and any interventions/support provided following use will not be ethnicity specific so should not stereotype individuals/communities. Additionally, although this category is broken down further, only a small amount of further detail is captured and numbers were too small to break down further for this specific analysis.*

Also, I note that the model is better at correctly identifying those at low risk than those at high risk. Is this model therefore best able to direct resources to those who most need it? Or will it be used instead to prohibit access to certain groups who are deemed low risk?
*A high proportion of those identified as low risk do not become overweight/obese and a higher proportion of those identified at high risk go on to become overweight/obese. This also improves as the child gets older and more information is available to predict risk. In resource poor settings, this can be used to target resources at those deemed at high risk. Additionally, the benefit of the different stages at which this model can be applied is that the levels of intervention can be varied dependent on child’s risk status. The reviewer raises a very valid point about potentially using the model to prohibit access of support to groups identified as low risk and we do not think the model should be used that way if those groups need the extra support. We have added this point to our discussion section (lines 351-356 in the clean and 356-360 in the tracked version).*

Discussion
I note that in your discussion you talk about the prevalence of obesity in deprived areas. But I come back to where your model seems strongest; that is in identifying those at low risk. Therefore, what are its practical uses in these settings?
The model seems to have a much higher specificity (85.3%) than sensitivity (59.6%). Is sensitivity what we should really be striving for?

*Interventions for childhood overweight and obesity are generally focussed on behavioural and lifestyle changes related to physical activity and diet. The potential harms to the individual of a behavioural intervention are likely to be lower than that of a clinical intervention. However, a successful cost-effective intervention for childhood overweight and obesity does not exist.*

*We are in agreement that sensitivity and specificity are both important. We have suggested a risk threshold that we feel balances all the parameters and also takes in consideration the population identified as at risk. However, this is a suggestion and other risk thresholds may be used. As stated in the discussion, we have categorised risk as low risk (0-20%, green), medium risk (20-30%, yellow) and high risk (≥30%, orange) in the website we have developed for the tool.*

Reviewer #2: Dear Authors,

Thank you for submitting the following manuscript to PLOS Global Public Health:
Manuscript ID: PGPH-D-21-00446
Type of manuscript: Research Article
Title: Childhood overweight and obesity at the start of primary school: external validation of pregnancy and early-life prediction models
Authors: Nida Ziauddeen, Paul J. Roderick, Gillian Santorelli, John Wright, Nisreen A. Alwan.

Unfortunately, at this stage, the manuscript does not qualify for being Peer Reviewed. It seems that the authors performed considerable work in order to collect the data of so many participants; however, the analysis appears to be too simple considering the sample size. Moreover, other shortcomings need to be corrected and clarified:

1. The same research was published elsewhere. As found on the following link: <https://jech.bmj.com/content/74/Suppl_1/A26.1>. On the 10th of September 2020, as an oral presentation, which contradicts the PLos Journal policy. Please justify.
2. The Introduction is also a repetition of the same published paper (<https://jech.bmj.com/content/74/Suppl_1/A26.1>.)
*Based on journal editor comments above, we have not responded to these comments which relate to a published conference abstract.*

3. In the results section,
- Figure 1: needs more clarification. Kindly add a caption or words to the figure to help guide the reader and ease the reading of the figure.

*Thank you for this suggestion. We have now added a caption that we hope eases the reading of the figure (lines 206-208 in the clean and 209-211 in the tracked version).*

- Table 2 and Table 3: the title needs to be more representative of the data provided; specifically, suggesting adding “predictor” at 4-5. It is unclear because the authors reported the 1&2 years old on the table but referred to 4-5 years old in the title.
*The outcome of childhood overweight and obesity at 4-5 years is predicted using predictor data up to 2 years. We have added “using predictor data at booking, birth and early life (~ 1 and 2 years)” to the titles for both Tables 2 and 3. We hope this helps clarify the title (lines 236-238/246-248 in the clean and 239-241/250-252 in the tracked version).*

4. The Discussion section:
- The authors only reported the results, and no supporting studies were included.
- In the strengths and limitations, it is advised to be more precise in reporting the strengths and limitations of the study.
*Thank you, we have now updated the discussion section to include supporting studies (lines 260-290 in the clean and 264-294 in the tracked version) and updated the strengths and limitations section (lines 293-314 in the clean and 297-318 in the tracked version).*

5. The conclusion of the study is unclear and needs improvement.
Suppose the authors feel that they can adequately address all the issues and considerably improve the quality of this study by performing a more in-depth analysis. In that case, they can resubmit their manuscript as a new submission.
Wish you every success if you choose to submit it elsewhere.
Good luck and best wishes

*Thank you, we have updated the study conclusion (lines 359-361 in the clean and 364-366 in the tracked version).*

Reviewer #3: Please see attached pdf for comments

This manuscript details the performance of a previously developed model for predicting overweight and obesity risk when applied to a new dataset. This kind of external validation is an important and often overlooked part of model building and if the comments below are sufficiently well addressed then I believe this work is worthy of publication. The manuscript is mostly clear and well written. However, I believe this work could be improved in a number of ways. My main suggestions and some more minor comments are detailed below.

Main suggestions:

1. The model that is being validated should be described in more detail. While the

model considered has already been described in the literature, it is such a central feature of this study that I believe there needs to be a description of the model structure in the methods section. Currently it is not described at all.

*Thank you for this suggestion. We have added more detail in the introduction section by briefly outlining the model development process and linked to the published paper on model development in this section as well (lines 95-109 in the clean and 96-111 in the tracked version).*

2. There should be greater contextualisation of the model. There is some discussion of other models in the introduction but only the predictors used in other models are discussed. It would be informative to discuss the methods used. Information of the performance of these models would also provide important context for the results presented in this work.

*Thank you, we have added further detail on methods used and model performance in the introduction (lines 76-88 in the clean and 77-89 in the tracked version) and have also included more detailed consideration of model performance on external validation of other models for predicting childhood overweight/obesity compared to our study in the discussion (lines 260-290 in the clean and 264-294 in the tracked version).*

3. It could be useful to discuss the utility of the model in practice. Having shown that the model performs to a certain level on external data, could this model then be used in practice to identify at risk children? What methods are currently used? While I appreciate this is a largely methods-focused paper, including these ideas in the discussion would provide more insight into the utility of this model.

*The research team based at the University of Southampton are currently running a feasibility study on the acceptability and usability of the tool in clinical practice. Current clinical practice in the UK uses health professional clinical judgement and usually provides support later when child is already overweight/obese or further on the path to being overweight/obese. We have now included consideration of this in the discussion (lines 344-356 in the clean and 348-360 in the tracked version).*

Minor points:

1. I found the term “development model” a bit confusing, since the same model was used in both cases and it was the validation method that was different. If the term “development model” is used then I think it needs to be defined somewhere (in terms of what data it was trained and validated on). Alternatively it might be clearer to rephrase the comparison in terms of the validation schemes (e.g. cross validation vs external validation).

*Thank you, we have rephrased the sentence to identify this as the process as follows “The AUC on external validation was comparable to that on development at all stages (booking, birth, ~1 year and ~2 years).” (lines 222-224 in the clean and 225-227 in the tracked version).*

2. In the model considered, the response variable (BMI percentile) is dichotomised and then I believe the model is trained on these binary outcomes. This dichotomisation results in a loss of information — could it be more effective to model BMI percentile directly? This kind of dichotomisation of response variables is often warned against,largely for this reason. This is something that could be discussed in the discussion.

*Thank you. Clinical prediction models are mainly of two types – diagnostic and prognostic. A diagnostic model is used to predict the probability of developing a particular disease or condition and a prognostic model aims to predict the probability of a particular future outcome/event in individuals with a certain condition or disease. As this work is used to predict the probability of developing overweight/obesity, the outcome is necessarily dichotomised.*

*All continuous predictor variables have been retained as continuous to avoid loss of information through categorisation.*

3. Related to the previous point, I don’t think it specifies anywhere how the model was previously validated. I think this should be included.

*The method of internal validation has now been added to the introduction section where more details of the model has been provided (lines 108-109 in the clean and 111 in the tracked version).*

4. Line 58 - “Prediction models provide more accurate risk estimates compared to more subjective predictions” this is quite a broad statement supported by a fairly specific reference. I would suggest finding additional relevant references and potentially qualifying this statement.

*We have added an additional reference to support this statement (line 71 in the clean and 72 in the tracked version). This is difficult to quantify due to variation in clinical assessment/diagnosis criteria for different diseases, the availability of prediction models and limited studies that have compared the two.*

5. Lines 61-62. This sentence needs to be edited slightly, something like “Prediction models generated using routinely collected data are easier to apply in clinical practice as [they only utilise] data…”

*Thank you, this has now been updated as suggested (line 74 in the clean and 75 in the tracked version).*

6. Line 128 needs a semicolon after (booking)

*Thank you, this has now been updated as suggested (line 162 in the clean and 165 in the tracked version).*

7. Line 174 need the word “mean” at the beginning of this sentence

*Thank you, this has now been added as suggested (line 209 in the clean and 212 in the tracked version).*

8. Line 176 “Nearly two-thirds of women reported being never smokers.” should this be three-quarters?

*Thank you, this should been three-quarters and has been updated (line 211 in the clean and 214 in the tracked version).*

9. Table 1 - Needs a clearer explanation as to what the third column (%>91st centile BMI). How can the percentage of never, ex, and current smokers above the 91st centile all be greater than 9? It seems likely that I’ve misunderstood the meaning so clarification would be useful.

*Thank you, we have updated this to state “% of children ≥91st centile BMI”. This column presents the percentage of children who are ≥91st centile BMI (overweight/obese) for each category for the categorical variables. For example, 17.2% of children whose mothers were current smokers during pregnancy were overweight/obese compared to 14% of children whose mothers were never smokers. We have also added a sentence to explain this in the text (lines 216-218 in the clean and 219-221 in the tracked version).*

10. Lines 186-188. “The AUC at development was 0.66 (95% confidence intervals (CI) 0.65 to 0.67) compared to 0.64 (95% CI 0.62 to 0.66) on external validation.” This sentence needs to state what stage it refers to (while the next sentence specifies ~2 years).

*Thank you, we have added “at booking” to this sentence to specify the stage (line 224 in the clean and 228 in the tracked version).*

11. Figure 2 - if the observations are grouped by each 10th of predicted risk then why aren’t the points on the plot at every 10% on the x axis? If the observations are grouped by observed risk then the same question but for the y axis.

*Thank you. The wording of this was misleading for which we apologise. The figures are grouped into ten equal groups (by sample size) of predicted risk so the mean predicted risk increases across the groups from group 1 to group 10 but does not correspond to percentages on the axis. We have updated the wording in this section so it now reads “This was assessed for each tenth of predicted risk, ensuring 10 equally sized groups, by calculating the ratio of predicted to observed risk and plotting observed proportions versus predicted probabilities.” We hope this is clearer (lines 187-190 in the clean and 190-193 in the tracked version).*

12. Line …. - on lines 149-151 it is stated that discrimination is measured using AUC, however on lines 205-206 it is stated that “This indicates that the discrimination performance is highest at the ≥20% risk threshold”. As far as I understand, AUC does not depend on the binary classifier threshold—instead it is calculated from an ROC curve which itself is produced by varying the threshold over all possible values. Therefore, how can discrimination (quantified by AUC) be highest at a certain risk threshold?

*Thank you for pointing this out. We have reworded this sentence this to “This indicates that the optimal threshold for determining high risk is 20%.” (lines 244-245 in the clean and 247-249 in the tracked version).*

13. First paragraph of the “strengths and limitations” section. Paragraph lines 221-238. This paragraph mostly details the differences and similarities between the development and external validation cohorts. However, it provides little insight into whether these differences or similarities consitute strengths or limitations of this work. This should be added. The last sentence of this paragraph also seems fairly strong and could be qualified.

*We have now clarified that the differences between the cohorts is a strength of this analysis and qualified the last sentence (lines 309-314 in the clean and 313-318 in the tracked version).*
