# Supplementary material for: Childhood overweight and obesity at the start of primary school: External validation of pregnancy and early-life prediction models
Source: PLOS Glob Public Health. 2022 Jun 9;2(6):e0000258. doi: 10.1371/journal.pgph.0000258 (PMC10022097; doi:10.1371/journal.pgph.0000258)
Supplement: Attachment — Submitted filename: Response_to_reviewers_R2.docx [file pgph.0000258.s002.docx]

Response to reviewers

The reviewers have found your paper to be suitable for publication in PGPH. However, reviewer 1 has raised some minor issues that will need your attention:

They felt that the utility of the model, and its limitation needs to be explained more clearly with no hesitation.

*Thank you, we have amended this to be more clear.*

I have noticed the following issues after going through the manuscript, which must be addressed before it can be accepted for publication:

1. It appears lines 150-156 are repetitions of lines 130-137. Please, check this. You could merge some of the information in the former with the latter.

*Thank you for raising this. We would like to clarify that lines 130-137 refer to the outcome at 4-5 years measured as part of the National Child Measurement Programme and details of this was converted to BMI z-scores. Lines 150-156 refer to height and weight measured at 12 and 24 months of age as part of the Born in Bradford cohort and are predictors of the outcome. We have provided details on how this was measured here. We have amended to try to clarify.*

2. More importantly, the discussion section needs to be reworked on. It looks more like a results section. The detail statistics (e.g. OR, CIs) are not needed in this section. They should be removed, and the results discuss in the context of the existing literature. 
*The 95% CI reported in the discussion are from published literature and not this study. We have deleted these as suggested and discussed the results in the context of the existing literature. We have retained the AUC as this is necessary to compare/discuss model performance with existing literature. For example, an AUC of 0.7-0.8 is considered fair but as this a range, the differences in AUC on development and external validation cannot be discussed without the actual values if both are within this range as a higher value indicates higher discrimination.*
